# Mitochondrial oxidative stress and nitrate tolerance – comparison of nitroglycerin and pentaerithrityl tetranitrate in Mn-SOD^+/- ^mice

**DOI:** 10.1186/1471-2261-6-44

**Published:** 2006-11-08

**Authors:** Hanke Mollnau, Philip Wenzel, Matthias Oelze, Nicolai Treiber, Andrea Pautz, Eberhard Schulz, Swenja Schuhmacher, Kurt Reifenberg, Dirk Stalleicken, Karin Scharffetter-Kochanek, Hartmut Kleinert, Thomas Münzel, Andreas Daiber

**Affiliations:** 1 The 2nd Medical Clinic, Johannes Gutenberg-University, Mainz, Germany; 2 University of Ulm, Department of Dermatology and Allergology, Ulm, Germany; 3 Department of Pharmacology, Johannes Gutenberg-University, Mainz, Germany; 4 Central Laboratory Animal Facility, Johannes Gutenberg-University Mainz, Germany; 5 Actavis Deutschland GmbH, Langenfeld, Germany

## Abstract

**Background:**

Chronic therapy with nitroglycerin (GTN) results in a rapid development of nitrate tolerance which is associated with an increased production of reactive oxygen species (ROS). According to recent studies, mitochondrial ROS formation and oxidative inactivation of the organic nitrate bioactivating enzyme mitochondrial aldehyde dehydrogenase (ALDH-2) play an important role for the development of nitrate and cross-tolerance.

**Methods:**

Tolerance was induced by infusion of wild type (WT) and heterozygous manganese superoxide dismutase mice (Mn-SOD^+/-^) with ethanolic solution of GTN (12.5 μg/min/kg for 4 d). For comparison, the tolerance-free pentaerithrityl tetranitrate (PETN, 17.5 μg/min/kg for 4 d) was infused in DMSO. Vascular reactivity was measured by isometric tension studies of isolated aortic rings. ROS formation and aldehyde dehydrogenase (ALDH-2) activity was measured in isolated heart mitochondria.

**Results:**

Chronic GTN infusion lead to impaired vascular responses to GTN and acetylcholine (ACh), increased the ROS formation in mitochondria and decreased ALDH-2 activity in Mn-SOD^+/- ^mice. In contrast, PETN infusion did not increase mitochondrial ROS formation, did not decrease ALDH-2 activity and accordingly did not lead to tolerance and cross-tolerance in Mn-SOD^+/- ^mice. PETN but not GTN increased heme oxygenase-1 mRNA in EA.hy 926 cells and bilirubin efficiently scavenged GTN-derived ROS.

**Conclusion:**

Chronic GTN infusion stimulates mitochondrial ROS production which is an important mechanism leading to tolerance and cross-tolerance. The tetranitrate PETN is devoid of mitochondrial oxidative stress induction and according to the present animal study as well as numerous previous clinical studies can be used without limitations due to tolerance and cross-tolerance.

## Background

Although organic nitrates such as nitroglycerin (glyceryl trinitrate, GTN) have been used for over a century in the therapy of cardiovascular diseases such as stable and unstable angina [1] the underlying mechanisms of nitrate bioactivation and development of nitrate tolerance are not completely understood and are most likely multifactorial [2]. Previously, we found that 3 days of nitrate treatment doubled vascular superoxide (O_2_^-^) production [3] which was also found in human bypass vessels from nitroglycerin (GTN) treated patients [4]. The crucial role of oxidative stress for the development of nitrate and cross-tolerance has been repeatedly demonstrated in numerous cell culture [5,6], animal [7-10] and human studies [11-15]. Most beneficial effects of therapeutics on nitrate tolerance were based on antioxidative properties [16-18].

In 2002, the mitochondrial aldehyde dehydrogenase (ALDH-2), which is subject to an oxidative mechanism-based inactivation, has been identified as a GTN-metabolizing enzyme and a possible important component in the processes leading to tolerance [19]. This concept was supported by recent studies in ALDH-2 deficient mice (ALDH-2^-/-^) [20]. Our laboratory further substantiated this concept in an animal model of *in vivo *tolerance and extended previous observations by demonstrating that mitochondria are a major source of ROS formation in response to acute and chronic GTN challenges [21-23]. The importance of the ALDH-2 concept for clinical nitrate tolerance was proven by two independent clinical studies in Asian subjects with a point mutated, dysfunctional ALDH-2 [24,25].

In the present study, we sought to characterize the role of mitochondria as a source of superoxide formation for the development of *in vivo *nitrate tolerance in response to GTN. We also used the alternative organic nitrate, PETN, which has been described to cause no tolerance, to see whether lack of tolerance induction is associated with a decrease in ROS formation. We also addressed the initial steps underlying PETN protective effects which have been described to be based on induction of heme oxygenase-1 and formation of the potent antioxidant bilirubin [26,27].

## Methods

### Materials

PETN (with 80 % (w/w) lactose) was from Fluka (Buchs, Switzerland). For isometric tension studies, GTN was used from a Nitrolingual infusion solution (1 mg/ml) from G.Pohl-Boskamp (Hohenlockstedt, Germany). For induction of *in vivo *tolerance, GTN was used from a solution in ethanol (102 g/l) which was obtained from UNIKEM (Copenhagen, Denmark). L-012 (8-amino-5-chloro-7-phenylpyrido [3,4-d]pyridazine-1,4-(2H,3H)dione sodium salt) was purchased from Wako Pure Chemical Industries (Osaka, Japan). All other chemicals were of analytical grade and were obtained from Sigma-Aldrich, Fluka or Merck.

### Animals and in vivo treatment

All animal treatment was in accordance with the Declaration of Helsinki and with the Guide for the Care and Use of Laboratory Animals as adopted and promulgated by the U.S. National Institutes of Health and was granted by the Ethics Committee of the University Hospital Eppendorf and the University Hospital Mainz. We used male mice aged 7–10 months on a mixed genetic background (C57Bl/6 × 129/Ola). Experiments were performed with 14 wt and 14 Mn-SOD^+/- ^mice. Mn-SOD^+/- ^mice were generated according to a published procedure [28] in the laboratory of K. Scharffetter-Kochanek. The deficiency of the Mn-SOD activity was determined using a specific activity assay as described recently [28]. *In vivo *tolerance was induced by chronic infusion of mice with GTN in ethanol (25 μg/h, 60 nmol/min/kg for 4 d) by implanted micro-osmotic pumps (alzet, model 1007D, 0.5 μl/h for 7 d) from Durect Corp. (Cupertino, CA). Infusion of the solvent ethanol served as a control. We also infused mice with PETN in DMSO (35 μg/h, 60 nmol/min/kg) or the solvent alone. After this period, the animals were sacrificed and aorta as well as hearts were subjected to further analysis. The detailed protocol was recently published [23]. Wistar rats were either infused with ethanol (1 μl/h for 4 d) as the solvent control or GTN in ethanol (6.6 μg/kg/min for 4 d) to induce tolerance as previously published [16,22]. In These Wistar rats we used micro-osmotic pumps (alzet, model 2001, 1 μl/h for 7 d) from Durect Corp. (Cupertino, CA).

### Isometric tension studies

Vasodilator responses to GTN, PETN, ISDN and ACh were assessed with endothelium-intact isolated murine aortic rings mounted for isometric tension recordings in organ chambers upon preconstriction with prostaglandin F_2α_, as described previously [29]. It is important to note that the order of subsequent concentration-relaxation-curves was ACh, ISDN, GTN (in GTN *in vivo *treatment group) and ACh, PETN, GTN (in PETN *in vivo *treatment group). This order is important to explain the phenomenon of tachyphylaxis induced during tension studies in the organ baths.

### Detection of oxidative stress in isolated heart mitochondria

Isolated mitochondria were prepared from mouse hearts according to a previously published protocol and ROS formation was detected by L-012 (100 μM) ECL as recently described [22,23,30]. Briefly, hearts from mice were homogenized and the 20,000 g pellet was resuspended in Tris buffer. Mitochondrial suspensions were diluted to a final protein concentration of 0.1 mg/ml in 0.5 ml of PBS buffer containing L-012 (100 μM). ROS production was detected after stimulation with succinate (4 mM final concentration). Mitochondria from rat heart were prepared by a similar procedure and ROS formation was detected by L-012 ECL as published [31]. Effect of bilirubin on ROS formation was tested at concentrations of 0.25–25 μM from a 1 mM stock in DMSO.

### ALDH-2 dehydrogenase activity in isolated mouse heart mitochondria

The activity of ALDH-2 in isolated mitochondria was determined by measuring the conversion of 2-hydroxy-3-nitro-benzaldehyde to 2-hydroxy-3-nitro-benzoic acid using a modified published protocol [22]. The mitochondrial suspension was diluted to approximately 1 mg/ml protein in PBS and 2-hydroxy-3-nitro-benzaldehyde (100 μM) was added and the samples were incubated for 30 min at 37°C. 100 μl of each sample were subjected to HPLC analysis.

### Effects of GTN and PETN on HO-1 mRNA expression in cultured EA.hy 926 cells

Human endothelial cells EA.hy 926 cells [32] were grown in Dulbecco's modified Eagle's medium (Sigma) with 10% fetal calf serum, 2 mM L-glutamine, 1 mM sodium pyruvate, 100 IU/ml penicillin, 100 μg/ml streptomycin, and 1× HAT (hypoxanthine, amethopterin/methotrexate, thymine) [33]. Confluent cells (6-well plates) were incubated with either DMSO (0.1 %), 10 μM PETN, 50 μM PETN or ethanol (0.1 %), 10 μM GTN, 50 μM GTN for 12 h. mRNA expression of HO-1 was analyzed with quantitative real-time RT.

Briefly, total RNA from EA.hy926 cells was isolated according to the manufacturer's protocol (RNeasy Mini Kit; Qiagen, Hilden, Germany). 0.5 μg of total RNA was used for real-time RT-PCR analysis with the QuantiTect™ Probe RT-PCR kit (Qiagen) in 25 μl reactions in a 96-well spectrofluorometric thermal cycler (iCycler, Bio-Rad, München, Germany). Real-time qRT-PCR was performed according to the manufacturer's recommendations using the oligonucleotides listed below (all from MWG-Biotech, Ebersberg, Germany).

HO-1

sense AGGCCAAGACTGCGTTCCT

antisense GGCTCTGGTCCTTGGTGTCAT

probe CTCAACATCCAGCTCTTTGAGGAGTTGCAG

GAPDH

sense CCCATGTTCGTCATGGGTGT

antisense TGGTCATGAGTCCTTCCACGATA

probe CTGCACCACCAACTGCTTAGCACCC

Each experimental reaction was performed in triplicate. All primer/probe sets had efficiencies of 100% (± 10%). The comparative Ct method was used for relative mRNA quantification [34]. Gene expression was normalized to the endogenous control, GAPDH mRNA, and the amount of target gene mRNA expression in each sample was expressed relative to that of control.

### Statistical Analysis

Results are expressed as mean ± SEM. One-way ANOVA (with Bonferroni's or Dunn's correction for comparison of multiple means) was used for comparisons of vasodilator potency and efficacy, L-012-derived chemiluminescence, ALDH-2 activity as well as HO-1 mRNA expression. The EC_50 _value for each experiment was obtained by log-transformation. P values < 0.05 were considered significant. * indicates significance vs. solvent control.

## Results

### Effect of heterozygous deficiency in mitochondrial superoxide dismutase (Mn-SOD^+/-^) on vasodilator responses in response to organic nitrate treatment

**Figure 1:**
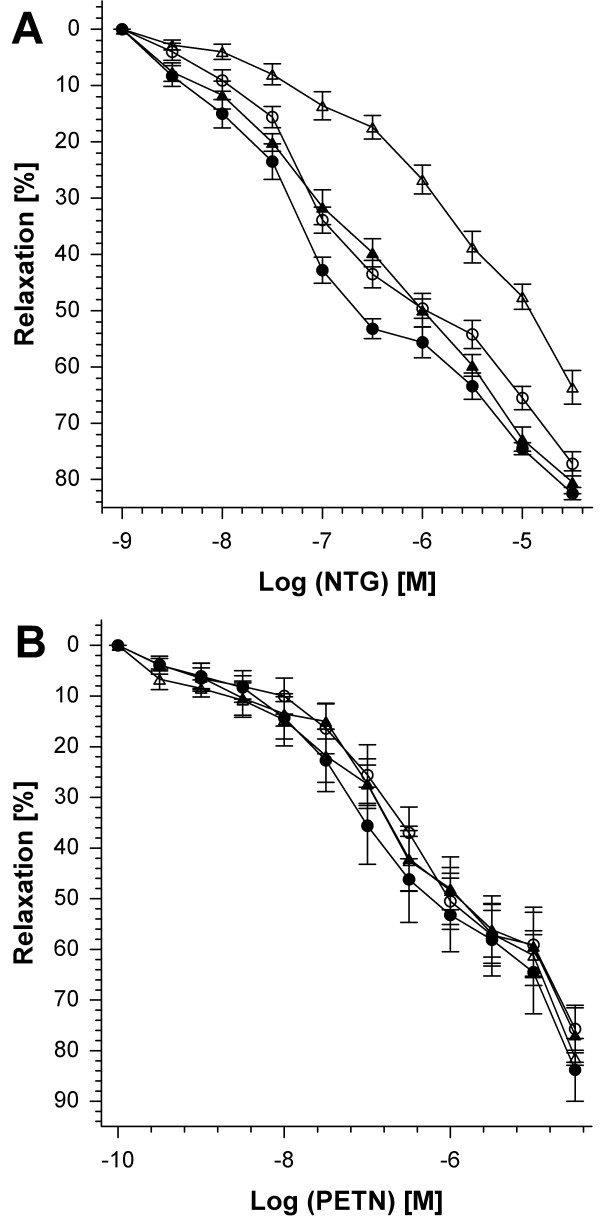
**Vasodilator responses of isolated aortic vessel segments upon chronic treatment of wild type and Mn-SOD^+/- ^mice with GTN or PETN**. (**A**) Concentration-relaxation curves for GTN (10^-9 ^to 10^-4.5 ^M) in vessels from wild type and Mn-SOD^+/- ^mice upon treatment with ethanol or GTN in ethanol. The symbols are closed circles (ethanol infused wild type), closed triangles (GTN infused wild type), open circles (ethanol infused Mn-SOD^+/-^) and open triangles (GTN infused Mn-SOD^+/-^). Data are mean ± SEM of 7–10 independent experiments. (**B**) Concentration-relaxation curves for PETN (10^-10 ^to 10^-4.5 ^M) in vessels from wild type and Mn-SOD^+/- ^mice upon treatment with DMSO or PETN in DMSO. The symbols are closed circles (DMSO infused wild type), closed triangles (PETN infused wild type), open circles (DMSO infused Mn-SOD^+/-^) and open triangles (PETN infused Mn-SOD^+/-^). Data are mean ± SEM of 6–8 independent experiments.

We here show for the first time data from isometric tension studies indicating that a partial (50 %) deficiency in Mn-SOD and accordingly increased basal mitochondrial ROS levels result in a more pronounced clinical tolerance. This phenomenon was characterized by impaired vasodilator potency of GTN and a significant right-shift of the concentration-relaxation curve (Figure 1A). Similar effects were observed for the concentration-relaxation curves of the endothelium-dependent vasodilator ACh as well as the nitrovasodilator isosorbide dinitrate (ISDN), indicating mild but not significant cross-tolerance to these compounds in GTN-treated Mn-SOD vessels (see Table 1). It is important to note that the GTN *in vivo *dose was adjusted as compared to previous studies to cause no severe tolerance in wild type control mice. Accordingly, GTN *in vivo *treatment of wild type mice with this low nitrate dose resulted in mild tolerance to GTN but obviously no cross-tolerance to ACh and ISDN (Table 1). In contrast, PETN *in vivo *treatment resulted in no clinical tolerance and we observed normal PETN and ACh potencies in vessels from wild type and Mn-SOD^+/- ^mice (Figure 1B, Table 1). Nevertheless, the PETN concentration-response-curve which was performed prior to the GTN concentration-response-curve resulted in decreased potency of GTN (Table 1). This indicates that *in vitro *incubation with the concentration of PETN that caused 100 % relaxation (ED_100 _= 31.6 μM) induced *in vitro *tolerance (tachyphylaxis) to the finally applied nitrate GTN. The GTN potency in this subsequent concentration-relaxation-curve was shifted by more than one log as compared to normal GTN potency in mouse aorta (see wild type in Table 1).

**Table 1:** Vasodilator potency of ACh, GTN, PETN and ISDN in vessels from wild type or Mn-SOD^+/- ^mice upon chronic treatment with GTN or PETN.

	Potency, EC_50 _(-log M) ^a^			
	
*In Vivo *Treatment	ACh	PETN	GTN	ISDN
WT/EtOH	6.97 ± 0.2 (n = 7)	n.d.	6.98 ± 0.1 (n = 9)	4.44 ± 0.1 (n = 4)
WT/GTN	7.05 ± 0.2 (n = 8)	n.d.	6.44 ± 0.1 (n = 7)*	4.55 ± 0.1 (n = 4)
Mn-SOD^+/-^/EtOH	7.16 ± 0.1 (n = 10)	n.d.	6.68 ± 0.1 (n = 8)	4.38 ± 0.1 (n = 4)
Mn-SOD^+/-^/GTN	6.86 ± 0.1 (n = 9)	n.d.	5.77 ± 0.1 (n = 10)*^#^	4.17 ± 0.1 (n = 4)*

WT/DMSO	6.89 ± 0.1 (n = 7)	6.82 ± 0.3 (n = 6)	5.64 ± 0.2 (n = 7)	n.d.
WT/PETN	7.26 ± 0.0 (n = 5)^§^	6.57 ± 0.2 (n = 6)	5.97 ± 0.3 (n = 6)	n.d
Mn-SOD^+/-^/DMSO	7.12 ± 0.1 (n = 7)	6.55 ± 0.2 (n = 7)	5.70 ± 0.2 (n = 7)	n.d.
Mn-SOD^+/-^/PETN	7.30 ± 0.1 (n = 8)^§^	6.37 ± 0.1 (n = 8)	5.71 ± 0.1 (n = 8)	n.d

^a ^*, p < 0.05 vs. WT/EtOH and ^#^, p < 0.05 vs. Mn-SOD^+/-^/EtOH.

^§^, p < 0.05 vs. WT/DMSO and^$^, p < 0.05 vs. Mn-SOD^+/-^/DMSO.

n.d. means not determined.

### Effect of heterozygous deficiency in mitochondrial superoxide dismutase (Mn-SOD^+/-^) and bilirubin levels on mitochondrial oxidative stress in response to organic nitrate treatment

**Figure 2:**
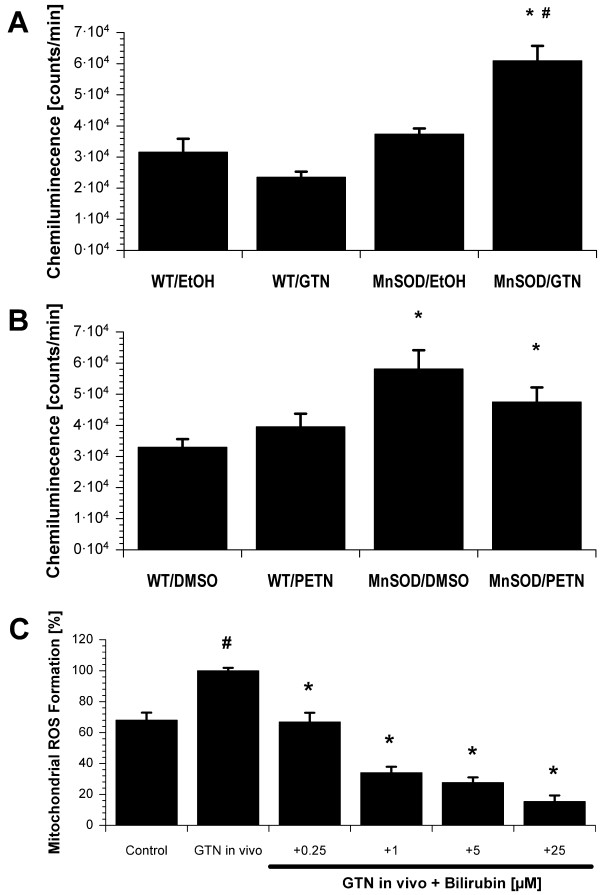
**Mitochondrial ROS formation upon chronic treatment of wild type and Mn-SOD^+/- ^mice with GTN or PETN and effects of bilirubin**. (**A**) Mitochondrial ROS formation was stimulated with succinate (2 mM) and measured by L-012 (100 μM) ECL in hearts from wild type and Mn-SOD^+/- ^mice upon treatment with ethanol or GTN in ethanol. Data are mean ± SEM of 4 independent experiments. * P < 0.05 vs. WT/EtOH and ^# ^P < 0.05 vs. MnSOD/EtOH. (**B**) Mitochondrial ROS formation was stimulated with succinate (2 mM) and measured by L-012 (100 μM) ECL in hearts from wild type and Mn-SOD^+/- ^mice upon treatment with DMSO or PETN in DMSO. Data are mean ± SEM of 16 independent experiments. * P < 0.05 vs. WT/DMSO. (**C**) Bilirubin efficiently decreased mitochondrial ROS (L-012 ECL) in response to GTN *in vivo *treatment of Wistar rats. Data are mean ± SEM of 6–11 independent experiments. ^# ^P < 0.05 vs. control, * P < 0.05 vs. GTN *in vivo *group.

In accordance to the vascular responses, mitochondrial ROS was not increased but rather decreased in PETN-treated Mn-SOD^+/- ^mice (Figure 2B). This was an important finding with respect to our recent observation that mitochondrial ROS levels were significantly increased upon GTN *in vivo *treatment of Mn-SOD^+/- ^mice [23] which was reproduced with the present findings (Figure 2A). Since the applied GTN dose was low, wild type mitochondria showed no increased ROS formation in response to GTN *in vivo *treatment which could be best explained by antioxidant properties of the small amounts of the solvent ethanol. Ethanol in turn could interfere with GTN bioactivation by the formation of the natural substrate and competitive inhibitor of bioactivation acetaldehyde.

Mitochondrial ROS were increased by almost 50 % in response to GTN *in vivo *treatment of Wistar rats (Figure 2C). Bilirubin concentration-dependently decreased the chemiluminescence signal in mitochondria from GTN-treated Wistar rats indicating its potent scavenging properties for GTN-derived ROS (Figure 2C).

### Effect of heterozygous deficiency in mitochondrial superoxide dismutase (Mn-SOD^+/-^) on mitochondrial ALDH-2 activity in response to organic nitrate treatment

GTN *in vivo *treatment decreased the redox-sensitive ALDH-2 activity in isolated heart mitochondria from Mn-SOD^+/- ^mice (30.4 ± 1.5 in ethanol group vs. 21.4 ± 1.5 in GTN group, n = 25–27, p < 0.001) whereas PETN slightly (although not significantly) increased the ALDH-2 activity (39.8 ± 3.3 in DMSO group vs. 42.3 ± 4.4 in PETN group, n = 9, p = 0.647).

### Effects of GTN and PETN on HO-1 mRNA expression in cultured EA.hy 926 cells

**Figure 3:**
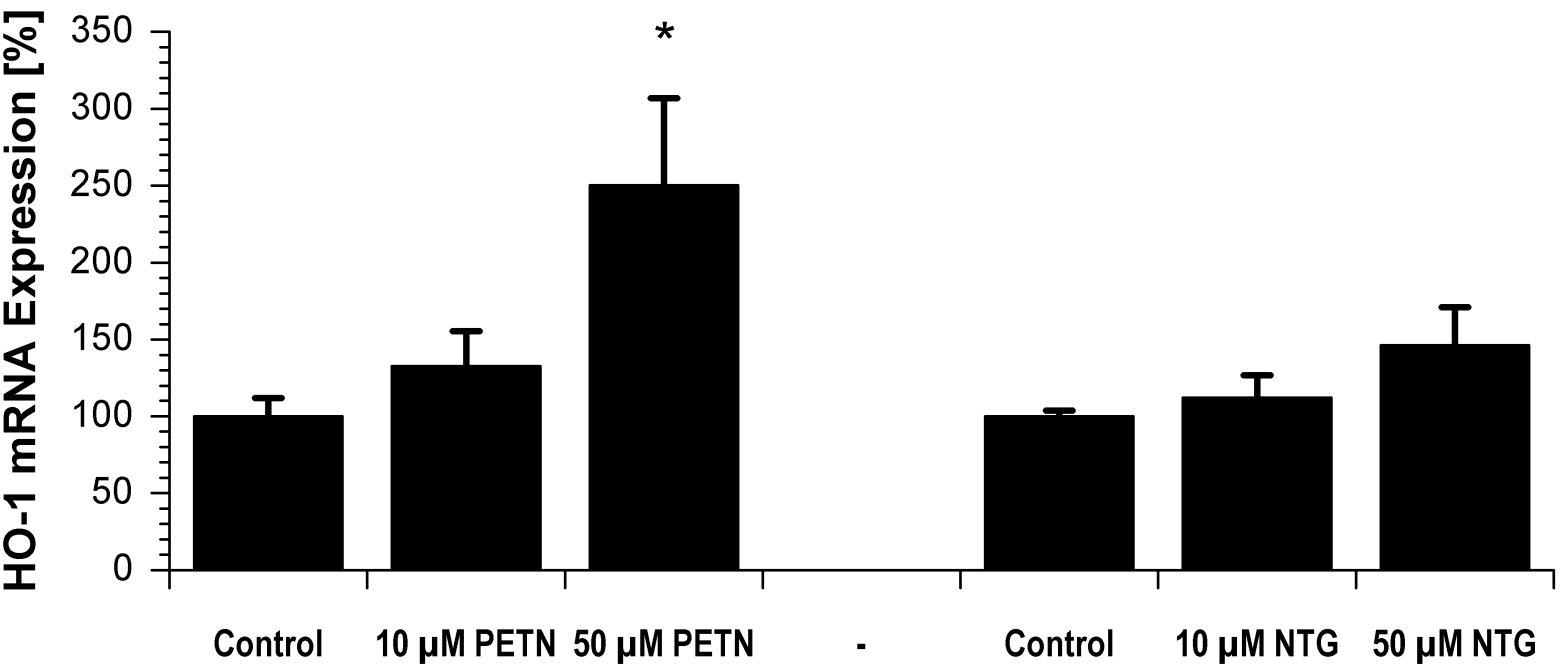
**HO-1 mRNA expression in EA.hy 926 cells in response to PETN or GTN treatment**. Confluent cells were incubated with the solvent (DMSO or ethanol) or PETN as well as GTN for 12 h. PETN but not GTN resulted in a significant increase in HO-1 mRNA. Data are mean ± SEM of at least 3 independent experiments. * P < 0.05 vs. control.

PETN concentration-dependently increased the HO-1 mRNA levels in EA.hy 926 cells resulting in a significant increase at 50 μM (Figure 3). In contrast, GTN only induced a minor increase in HO-1 mRNA which did not reach significance (Figure 3). Similar differences have previously been demonstrated between the PETN trinitrate metabolite PETriN and ISDN [26,27].

## Discussion

### Nitrate tolerance and the "oxidative stress concept"

Previous studies have demonstrated that nitrate tolerance in response to GTN *in vivo *treatment is a multifactorial phenomenon [2]. The "oxidative stress concept" in the setting of nitrate tolerance was established by our group [3] and refined during the last couple of years [4,23]. In essence, the concept consists of increased superoxide formation in response to nitrate treatment which decreases NO bioavailability, leads to peroxynitrite formation [8], NOS uncoupling [35] and impairs NO/cGMP signaling [4]. Moreover, oxidative inhibition of prostacyclin synthase [18] as well as mitochondrial ALDH activity [22] may present other key events in the development of nitrate tolerance. In the present study we demonstrate that low dose GTN for 4 d causes tolerance (indicated by a significant right-shift in GTN concentration-relaxation-curve) in Mn-SOD^+/- ^mice but has only minor effects on GTN potency and efficacy in wild type mice (Figure 1A). Also mild cross-tolerance to ACh and ISDN was observed (Table 1). In contrast, PETN which has been reported to possess protective effects, caused no tolerance (Figure 1B and Table 1).

### Inhibition of ALDH-2 and mitochondrial ROS formation

**Figure 4:**
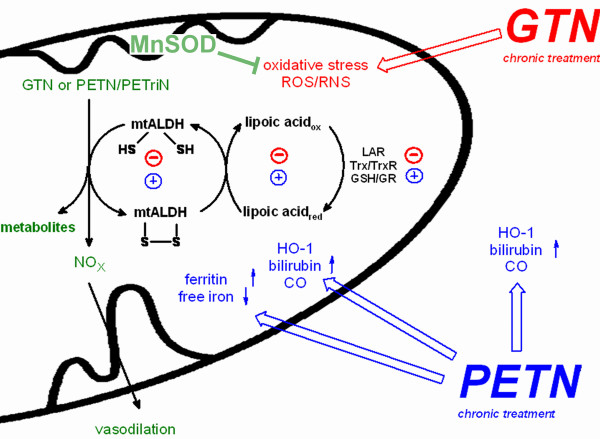
**Proposed mechanism for organic nitrate bioactivation, induction of oxidative stress and protective effects**. Highly potent organic nitrate (tri- and tetranitrates) are bioactivated by mitochondrial ALDH. The bioactivation (reduction cycle) requires two thiol groups at the active site of the enzyme which are oxidized to the disulfide during the conversion which yields the denitrated metabolite and an NO_x _species that is similar to NO. Enzymatic activity is restored by reduced lipoic acid/lipoamide which is recycled by lipoamide reductase (LAR), thioredoxin/thioredoxin reductase system (Trx/TrxR) or glutathione/glutathionereductase system (GSH/GR). GTN leads to mechanism-based inactivation of the enzyme but also triggers mitochondrial oxidative stress which may directly inactivate mtALDH, deplete dihydrolipoic acid or interfere with its reductases. In contrast, PETN induces HO-1 which by breakdown of porphyrins yields the potent antioxidant bilirubin, the anti-inflammatory compound CO and induces ferritin, another protective enzyme which decreases free iron and prevents Fenton-type reactions.

Recently, we have identified the mitochondria as a major source of oxidative stress in tolerant animals [16,21,22]. These results are in accordance with the observation that the mitochondrial ALDH provides an important bioactivation pathway for GTN [19,20,24,25] and is redox-sensitive [36,37] (see scheme in Figure 4). These facts provide a new link between oxidative stress and impaired GTN bioactivation and accordingly development of tolerance: Mitochondrial ROS oxidatively inactivate the ALDH-2. The involvement of mitochondria in the development of nitrate tolerance was previously postulated by the "thiol concept" of Needleman and coworkers [38,39]. An interesting link between mitochondrial oxidative stress triggered ALDH-2 inhibition and activation of vascular NADPH oxidases could be the accumulation of toxic aldehydes (as observed in ALDH-2^-/- ^mice). It was reported that low concentrations of 9-hydroxynonenal may stimulate whereas high concentrations may inhibit NADPH oxidase activity and/or expression [40]. In the present study we could demonstrate that GTN treatment resulted in an increase in mitochondrial ROS and a decrease in ALDH-2 activity in the Mn-SOD^+/- ^mice whereas PETN did not significantly alter these parameters (Figure 2 and Results).

### Diversity of organic nitrates with respect to induction of oxidative stress and clinical tolerance

The tetranitrate PETN was previously shown to cause no induction of clinical tolerance and vascular oxidative stress [12,14,17]. The beneficial properties of PETN were explained by induction of the antioxidative proteins ferritin and HO-1 [26,27] which may prevent oxidative stress and protect the vasculature from oxidative damage [2] and thereby mimic the antioxidant principle of compounds such as hydralazine [16]. HO-1 has been demonstrated to be a major protective and antioxidative principle in numerous therapeutic interventions [41,42]. The underlying mechanism of this protection is thought to be based on the breakdown of porphyrins to yield the potent antioxidant bilirubin [43-45] and the anti-inflammatory compound carbon monoxide [46,47] which is a weak activator of soluble guanylyl cyclase [48]. This is of special interest since HO-1 was reported to be localized within mitochondria [49] and thereby could directly affect nitrate-induced mitochondrial ROS formation and protect ALDH-2 from oxidative inactivation (see scheme in Figure 4).

There is growing body of evidence that ALDH-2 only bioactivates tri- and tetranitrates which show high potency in tension studies. During reduction of nitrates the dithiol groups at the active site of the enzyme form a disulfide bridge causing its inactivation. Dihydrolipoic acid is able to restore enzymatic activity (Daiber et al., unpublished observations) but needs reduction by appropriate enzymes. It is thought that GTN induces mitochondrial ROS formation which may contribute to oxidative inhibition of ALDH-2 activity and depletion of reduced thiols (such as dihydrolipoic acid) [38] thereby disrupting the physiological catalytic cycle (Figure 4). According to numerous reports in the literature PETN displays potent antioxidative properties which are probably based on the afore mentioned formation of bilirubin, CO and ferritin (Figure 4). In the present study we could demonstrate that bilirubin efficiently decreases mitochondrial oxidative stress in response to GTN treatment (Figure 2C). Moreover, we could reproduce previous results of Oberle et al. on PETN-triggered HO-1 induction and more importantly demonstrate that this inducing effect is not shared by GTN (Figure 3). This, however, could provide an explanation for the differences in tolerance induction by both organic nitrates.

One would expect that all NO donors induce HO-1 since this gene is heavily regulated by NO. However, previous publications have demonstrated that HO-1 inducing capacity is not shared by all NO donors [50]. It is unclear why ISDN and GTN do not induce HO-1. Especially, since HO-1 is regulated by at least 3 important transcription factors (NFκB, AP-1, Nrf2/Keap1) and induced by a huge number of different stimuli [51] providing many regulatory sites, which could explain the different properties of ISDN and GTN versus PETN. At this stage we have no hypothesis to explain these differences *in vitro*.

## Conclusion

The results obtained in Mn-SOD^+/- ^mice clearly indicate that mitochondrial ROS play an important role for the development/maintenance of nitrate tolerance. The results obtained with PETN indicate that organic nitrates differ markedly and that protective antioxidative properties (mediated by HO-1 and derived bilirubin) may compensate for harmful induction of oxidative stress sources.

## Abbreviations

**ACh: **acetylcholine, **ALDH-2: **mitochondrial aldehyde dehydrogenase, **ECL: **enhanced chemiluminescence, **GTN: **glyceryl trinitrate (nitroglycerin), **HO-1: **heme oxygenase-1, **ISDN: **isosorbide dinitrate, **L-012: **8-amino-5-chloro-7-phenylpyrido [3,4-d]pyridazine-1,4-(2H,3H)dione sodium salt, **Mn-SOD: **manganese superoxide dismutase (mitochondrial isoform), **Mn-SOD**^+/-^**: **heterozygous Mn-SOD deficiency, **PETN: **pentaerithrityl tetranitrate, **ROS: **reactive oxygen species.

## Competing interests

DS is an employee of Actavis Deutschland GmbH and TM received vascular research grants from Actavis Deutschland GmbH.

## Authors' contributions

1) HM: principal investigator, has made substantial contributions to conception and design of the study

2) PW: second principal investigator, has made substantial contributions to conception and design of the study

3) MO: performed animal surgery

4) NT: performed breeding of MnSOD^+/- ^mice

5) AP: performed mRNA measurements

6) ES: cultured EA.hy 926 cells

7) SS: performed incubations of EA.hy 926 cells

8) KR: was involved in animal care and critically revised the manuscript

9) DS: was involved in drafting and interpretation

10) KSK: was involved in drafting and interpretation

11) HK: was involved in drafting, interpretation and mRNA measurement

12) TM: was involved in drafting and interpretation and has made substantial contributions to conception and design of the study of data

13) AD: performed ROS and ALDH-2 measurements, was involved in drafting and interpretation and has made substantial contributions to conception and design of the study of data

Declaration: All authors read and approved the final manuscript

## Pre-publication history

The pre-publication history for this paper can be accessed here:


